# Supplementary material for: A LAMP assay for the rapid and robust assessment of Wolbachia infection in Aedes aegypti under field and laboratory conditions
Source: PLoS One. 2019 Nov 20;14(11):e0225321. doi: 10.1371/journal.pone.0225321 (PMC6867633; doi:10.1371/journal.pone.0225321)
Supplement: S1 Table — Top. Wolbachia wAlbB wsp LAMP assay (6-primer) [17]. Bottom. Aedes aegypti ITS1 LAMP assay (6-primer) [24]. (DOCX) [file pone.0225321.s001.docx]

**S1 Table**

**Table S1.** LAMP primers used in this study.

|  | Name | Sequence |
| --- | --- | --- |
| *Wolbachia w*AlbB *wsp* LAMP assay (6-primer) | *Wolbachia* *w*AlbB Surface Protein (*wsp*) gene target | TGCCTATCACTCCATACGTTGGTGTTGGTGTTGGTGCAGCATATATCAGCAATCCTTCAGAAGCTAGTGCAGTTAAAGATCAAAAAGGATTTGGTTTTGCTTATCAAGCAAAAGCTGGTGTTAGTTATGATGTAACCCCAGAAATCAAGCTTTATGCTGGTGCTCGTTATTTTGGTTCTTATGGTGCTAGTTTTAATAAAGAAACAGTATCAGCTACTAAAG |
|  | WSP.F3 | TGCCTATCACTCCATACGT |
|  | WSP.B3 | CTTTAGTAGCTGATACTGTTTCT |
|  | WSP.FIP | TGCTTGATAAGCAAAACCAAATCCTGGTGCAGCATATATCAGCAA |
|  | WSP.BIP | AGCTGGTGTTAGTTATGATGTAACCCACCATAAGAACCAAAATAACGAG |
|  | WSP.BLP | CCAGAAATCAAGCTTTATGCTGGTG |
|  | **WSP.FLP (new)** | **CTTTAACTGCACTAGCTTCTGAAGG** |
| *Aedes aegypti* *ITS1* LAMP assay (6-primer) |  |  |
|  | AE.ITS.F3 | CAAGGCACGTTACCMGG |
|  | AE.ITS.B3 | GCAGCACAACCACACGG |
|  | AE.ITS.FIP | GAGTGTCGCCGAGAGAAGCCGAGGACACTGCTGCACC |
|  | AE.ITS.BIP | TCGGACGCTCGTACGTACCGTCGAGCTTCGRCGASACA |
|  | AE.ITS.FLP | TGCGCGTGCCTCCGGGTGA |
|  | AE.ITS.BLP | CGAACGTGTCTGGCGTGTTCTG |
