# Supplementary material for: A LAMP assay for the rapid and robust assessment of Wolbachia infection in Aedes aegypti under field and laboratory conditions
Source: PLoS One. 2019 Nov 20;14(11):e0225321. doi: 10.1371/journal.pone.0225321 (PMC6867633; doi:10.1371/journal.pone.0225321)
Supplement: Attachment — Submitted filename: Response to Reviewers.docx [file pone.0225321.s003.docx]

***A LAMP assay for the rapid and robust assessment of Wolbachia infection in Aedes aegypti under field and laboratory conditions***

Response to reviewers

**Reviewer 1.**

1. **Too few samples**

We have increased the sample sizes for individuals in the 30 days at 26°C and the 10 days at 37°C categories (our most extreme, preservation-wise) to 18 to fill the gap. (See updated Figure 3).

2. **List number mosquitoes tested in methods**

These have been added (Lines 143-144)

3. **Clarify 5 and 6 primer LAMP usage**

Methods and results have been updated to reference construction of 5 and 6-primer sets (Lines 184-187, 236).

Figure S1 has also been updated to clearly communicate the use of 6 primers.

4. **Better address use of pool of 99 uninfected mosquitoes**

We do not present these results in figures because the only data to present are amplification times which are shown in the text.

‘Along the manuscript’, Lines 215-217 were updated to more clearly describe the motivation for the 99 pooled uninfected experiment. Lines 242-245 were also updated to more clearly distinguish the pooled sample experiment from the other experiments described in that section.

5. **qLAMP with pooled DNA is imprecise**

We thank the reviewer for their constructive feedback. It is likely low sample sizes contribute to the variable nature of the quantitation, but we also appreciate there are limits of detection resolution. Nevertheless we believe pooled detection methods are a viable strategy for monitoring large-scale *Wolbachia* releases—if carefully deployed as a component of an integrated monitoring scheme. Specifically we propose a two-tier system where LAMP quantitations are used in-field to rapidly quantify approximate frequencies across a large number of sites, with a central reference lab performing qPCR to follow up on key sites as identified through LAMP results. Rolling out of this process would benefit from an in-principle field demonstration and further standardisation and calibration.

We have updated our discussion to acknowledge further the quantitative imprecision of this pooled method, and to discuss in more detail how it could be used as part of a monitoring scheme (lines 330-341).

6. **cost of qPCR needs to be added**

The cost has been added at lines 353-354

**Reviewer 2.**

1. **Inappropriate use of the language of “improvement” on previous assay**

We thank the reviewer for drawing our attention to issues around the language used to describe what is novel about our work compared with the previously published (Bhadra) primers & assay process.

The 5-10 minute difference in amplification time assumes the use of our reagents & the Genie-III machine, which are integral to our assay. The original Bhadra assay calls for an amplification time of 90 minutes (compare under 25 minutes for ours). The trade-offs in terms of sequence specificity can be considered minimal for most purposes – the lack of a TaqMan-like probe is balanced by data output by Genie-III about amplification time and the annealing temperature of the product. This can be used to distinguish true amplifications from spurious amplifications – adequate for the majority of routine field monitoring uses.

Furthermore, our assay exceeds benchmarks of the Bhadra assay in some areas. Most importantly there is a substantial improvement in the ability to detect *Wolbachia* in mosquitoes stored at higher temperatures (such as in BG traps which are often only checked after several days). Bhadra et. al. detected only 40% of *Wolbachia* infected mosquitoes stored at 4°C (and none after week 2), and zero detection for samples stored at 37°C. We detect *Wolbachia* in 100% of individuals stored at 26°C for 1 month, and stored at 37°C for 10 days. And we detected *w*AlbB in 100% of mosquitoes left in water for 3 days prior to extraction.

As the reviewer has suggested, there will likely be many applications where the Bhadra assay remains more useful. Thus we agree that unqualified language of ‘improvement’ may not be appropriate. However, as we have outlined, our modifications do exceed the original assay on many metrics that are significant in field release monitoring. We have altered the reference to improving the Bhadra assay to instead reference ‘adapting and extending’ (line 105). We have also included some discussion explicitly comparing the two assays (lines 292-294).

2. **“we have developed” neglects dependence on previous study**

To clarify the intention behind these phrases, we have altered “The LAMP primer set we have developed” to read “The **modified** LAMP primer set we have developed” (lines 252-253), and altered “The LAMP assay we have developed” to read, “The **modified** LAMP assay we have developed (lines 298-299).

3. **Scatter in curve and lack of exact correlation**

We provide information about the samples used for concentration curves in the methods (lines 200-207). We also quantify the strength of correlation through the statistical regression results reported in lines 271-277, as well as through the 95% confidence intervals included in the Figure 1 graph.

We include further discussion about the imprecision issue in connection with the pooled DNA quantification (Reviewer 1 Q. 5, Reviewer 2 Q. 4).

4. **Infection frequency determination is imprecise**

We agree that there is some imprecision associated with frequency determination. Per our response to reviewer #1’s 5^th^ comment, we have acknowledged this in our discussion (lines 330-341).
